# Boston Soc. Med. Improvement

**Published:** 1855-03

**Authors:** Wm. W. Morland

**Affiliations:** Secretary


### EXTRACTS FROM THE RECORDS OF THE BOSTON SOCIETY FOR MEDICAL IMPROVEMENT.

BY WM. W. MORLAND, M. D., SECRETARY.

*Position in certain Gastric and Enteric Affections.*—Dr. COALE remarked, that the late frequency of cholera morbus and other similar affections, had given him an opportunity of testing, to a considerable extent, the efficacy of a certain practice of his, based upon observation made some time since, but which he felt wanted confirmation before suggesting it generally. He is convinced, from actual experiment, that persons affected with irritability of the stomach are much less liable to vomit if they lie on the right side than when they recline in any other position—particularly on the left side. The explanation is evident. While lying on the right side, any contraction of the stomach need not much affect its solid contents ; but, when lying on the left side, the contents are in the neighborhood of the cardiac orifice, and any contraction of the organ will force them more or less through this opening into the œsophagus ; thus, the difference between the two cases will be a simple eructation in the first, and vomiting in the second. This, Dr. C. has now tested in very many cases ; and by many experiments in some of them, varying the position to the increase or diminution of the nausea and vomiting. It may be urged in objection to the explanation, that a contraction of the stomach that would force the contents through the cardiac orifice, would produce vomiting at any rate. But the difference is this : the same amount of contraction, which, when the patient lies on the right side, throws off gas merely, when he is on the other may force a small portion of solid or fluid matter into the œsophagus, when reflex action is at once excited, and the whole stomach stimulated into action.

In treatment of cases of flatulence, and of what is commonly called "cramp colic," Dr. C. has found reclining on the right side beneficial. It lessens the vomiting—as first said—a frequent attendant in these cases; but, besides this, it gives a more ready escape to gas contained in the transverse colon. For example, suppose the trouble is a spasm, confining gas in the transverse or ascending colon, were the patient on the left side, and a relaxation of the spasm to occur, the gas is still kept behind the affected spot, for the distended intestine is not liable to take upon itself sufficient action to expel it. But, if the patient be on the right side, the gas then ascends and passes on to an unaffected part of the intestine, by which its escape is facilitated. At any rate, whatever may be the true explanation, Dr. C. is very confident of the correctness of his observations, and of the benefits resulting from this peculiarity of his treatment. He has not found any suggestion of this sort in treatises on the treatment of the diseases mentioned.

*Typhoid Fever—remarkable prevalence in one family. Remarks on certain Elements in the Causation and Propagation of Epidemic Diseases.*—Dr. Chas. E. Ware stated that he had recently seen several cases of typhoid fever, occurring in the same family, apparently depending upon some local cause. The house was situated in one of the healthiest parts of the city, and had everything within and about it to render it comfortable and healthful. A young girl of fourteen and one of the servants were taken ill, at about the same time, with the disease. Both died. A young boy was attacked with all the early symptoms, but was removed to the country, and, after a few days' illness, convalesced. Another—a servant, who came into the house during the sickness of the first—was only a few days in the house when she became sick and had typhoid. A young man of sixteen, cousin of the girl first attacked, and who was frequently, and for a long time, in the house visiting his cousin, had the disease. Immediately on the death of the first girl, the family shut up the house, and retired to the sea-side. There was not much typhoid about at the time, and there was none in the neighborhood.

Dr. Ware said that he had seen two or three instances, in the course of his practice, of typhoid having this local character. In one instance, seven boys, under twenty, the whole of the family except the mother, were sick together with well-marked typhoid. There were no other cases in the immediate neighborhood. It was an old wooden house which they occupied, but one which was clean and well-aired. In another instance, in a brick block of two houses, standing rather isolated, but near a stable, and not particularly well ventilated, there were eighteen cases of typhoid in the course of about three months, in the autumn. There was no unusual amount of the disease about at that time. In another instance, he had seen five cases in one family in the course of a season.

Dr. Bigelow, Sen., remarked that there seemed a probability that the acting cause, in Dr. Ware's cases was a *local* one ; it rarely happens that typhoid fever is taken by contact with persons who are removed to healthy localities. During twelve years at the Massachusetts General Hospital, several of the attendants and nurses were ill with typhoid fever ; it would seem as if they took it by contact with the other patients. The degree of contagious influence, said Dr. C., must be very inconsiderable, but is effective when the exposed individuals are, from any cause, peculiar susceptible ; the liability to communication of the disease in this way is not sufficient to deter persons from doing their duty to those ill with the fever, but is enough to lead to the exclusion of unnecessary visitors.

Dr. Minot asked of Dr. Ware if the condition of drains were known, in the locality referred to by him ?

Dr. Ware said there was nothing apparent to any of the senses, and the occupants of the house were unaware of anything out of order as to drainage.

[The house, as mentioned by Dr. Ware, is in an airy, dry situation ; is on one of the hill-streets, the declivity of which, although slight, is doubtless effective in favoring drainage ; and there has never been any effluvium perceptible ; during the previous summer, a thorough repair of the house was made, particularly of the cellar and lower story ; the cellar was enlarged, deepened slightly, cemented in parts, freed from any dampness, and no chance left for imperfection in drains, cesspools, or sinks. No illness of any sort occurred in the houses on each side of the one in which Dr. W.'s patients were ; nor, to our knowledge, any cases of typhoid fever in the street, other than those reported.—Secretary.]

Dr. J. B. S. JACKSON referred to a number of cases taking place, some years since, in one house, and that an entirely new one. Dr. JAMES JACKSON had charge of them.

Dr. Bigelow, Sen., spoke of the reported influence of filth in the causation and aggravation of epidemic disease; he thinks that it receives the blame thereof, in six cases out of seven, unjustly; it is well known that people who live in airy, healthy situations, yet have the same diseases; *density of population, in low-lying lands*, where occupation is cheap, is undoubtedly often an element in the causation of these affections, and in their increase. It is, added Dr. B., a fact that about as much filth exists in cities in healthy, as in sickly seasons.

Dr. Minot referred to the striking immunity from cholera in well-drained cities; he compared Boston, in this respect, favorably with others known as ill-drained.

Dr. J. O. STONE, of New York, who was present at the meeting, said that, in many of the side-streets of that city, the filth, during this season, had been, and still is, extreme; so much so, that it is impossible to drive close to the side-walks by reason of the accumulation; yet, in those very streets there was not a single case of cholera; filth had not seemed at all productive of the disease, and Dr. S. added that the city inspector thought it had had no effect in increasing the disease; dampness, said Dr. S., had an undoubted and decided influence in the production and continuation of cholera.

[May there not be supposed to be a great difference as to the possible action of accumulations of filth, arising from their *position?* A vast amount thereof might be innocuous, or nearly so, when lying in the *open and comparatively broad* streets; but were the same, or even a far less quantity, disposed in yards, narrow lanes, and closes; or, what is worse, in cellars, and near to or even *in* the rooms inhabited, would not there be an effect therefrom, and very palpable, also, upon those exposed? If disease has often, as none can doubt, been *generated* by these means, is it unlikely that existing epidemics are often aggravated by them? It is doubtless unwise to stir up all the hidden and long accumulated filth of a great city upon the first alarm of an approaching, or just appearing epidemic; for, perhaps, the very disturbance of these collections may then develope disease; "prevention being better than cure," the true policy, and indeed duty, of municipal and sanitory authorities is to *permit no accumulations likely*, even, to aid in producing or aggravating disease.—SECRETARY.]

Dr. PUTNAM referred to the remarkable exemption of the city of Lyons, France, which is surrounded by a river, from cholera.

Dr. J. B. S. Jackson spoke of the ease with which the city of St. Louis might be drained, from its position, &c.; he also alluded to the severity with which the city of Bangor, Maine, had been visited by cholera. Its largest part is high in situation.

Dr. Bethune remarked that there was, however, a large level space in both these latter cities, and if dampness be effective in causing choleraic disease, as it undoubtedly is, the fact of the vicinity of rivers to both cities may be of consequence.

Dr. Blake alluded to the very high situation of Quebec, yet cholera has there been found very destructive.

Dr. Bigelow, Sen., spoke of the former course of cholera, and also, in reference to Dr. Minot's suggestion as to the influence of efficient drainage, he said that Boston doubtless owed very much of her immunity from cholera to the arid, rocky soil; and drainage was much facilitated by the amount and frequency of declivity within the city.

Dr. Perry said that, in 1849, there were five fatal cases of cholera on St. James Street, in Roxbury, a high, airy, well-drained and dry situation; in 1832, he saw seven cases of cholera in the rear of Eliot Street, Boston; on examination of the premises, it was ascertained that a vault *was emptying itself into the well* used by the family.

In the cases at Roxbury, Dr. P. thought there was evidence in favor of the theory of communication of the disease by contagion; no imprudence in diet or otherwise was traceable, except in the *first* case, that of a servant, who, from imprudence, had diarrhœa, which was neglected; the different persons afterwards affected had been in close contact with, or had nursed each other; a child slept with one of the patients; a sister who attended the funeral of one patient, died of the disease soon afterwards; a woman who came from a distance and washed some clothes in the house, soon sickened and died.

Dr. C. E. Ware referred to the moving of the troops from Avingnon to Arles, in France; at the former place there had been cholera, at the latter none; it broke out, however, immediately on the arrival of the troops; the latter were then removed to Marseillies, and there the cholera raged. Either the disease moved along in the course of the troops, or else they carried it.

Dr. Bigelow spoke of the custom of moving troops from place to place, until they got free from epidemic disease. This plan is successful.

Dr. Homans, Sen., remarked that the course of *epidemic fever* had been very striking in the town in which he formerly practised. Typhoid fever prevails. There is a river with a fall of only four feet in six miles, and the adjacent grounds are, at times, overflowed by it. In July, "gastric fever" occurs; in the latter part of August and in the first part of September, typhoid fever begins to manifest itself. Dr. H. described the course and frequency of the fever during one year. Of eighteen persons in two houses, twelve had gastric affection, with fever; by the last of July, one person in nearly every house in the track of the fever had been affected; the disease followed the course of the river, then branched off around a large pond. In one house, there were seven ill in the time between September 1st and the middle of October; after that time, the disease went into houses situated upon hills. Sixty-eight or sixty-nine persons were attacked, in all. Brookfield, Mass., is the town referred to. In Oakham, which is the adjoining town, a physician, thirty years in practice *knew of no case of typhoid fever.*

*Poisoning by Strong Tincture of Aconite.*—Dr. PUTNAM reported a case in which most remarkable success followed the internal administration of iodine, as recommended in a recent French journal. Dr. REYNOLDS saw the patient about an hour after she had, by mistake, swallowed an ounce of strong tincture of aconite. Immediate vomiting ensued, and she was still vomiting; there was severe cramp; coldness of the whole surface, and especially of the extremities.

A solution was made of eight grains of hydriodate of potass and six grains of iodine in one quart of water. Of this a wineglassful was given, and the patient soon fell asleep. In half an hour she awoke in renewed distress; the solution was repeated; she again fell asleep, and, in the course of two hours, all formidable symptoms had disappeared.

Dr. Putnam remarked that, in the case of poisoning by aconite which he reported some months since, great benefit was derived from the administration of laudanum, after emesis induced by ipecac.

*Nov.* 13, 1854. *Falls from a great height—Remarkable Escape from Injury.*—Dr. COTTING, of Roxbury, Associate Member of the Society, related the following instance of escape from injury, after falling from a great height; and, in one, from a railway engine:

1. An Irishman, at work upon the steeple of the new Roman Catholic church, in Roxbury, fell from a height of fifty feet, by estimate, to the roof of the building, struck upon his hip and back, and slid to the eaves. No injury was sustained by him except some slight contusions; he returned to his work upon the same steeple *in two days afterwards.*

2. S. H., 16 years old, of stout frame, while attempting to disengage the hook of a hoisting apparatus in a store, lost his balance, and fell headlong through the scuttle, from the fifth story, fifty-four feet by exact measurement. When about half way down, he caught the rope with his left hand, and thus partially came into right position, feet downwards. Within ten or twelve feet from the lower floor, he caught the rope again with his right hand, and struck the floor upon his feet. He fell fainting into the arms of a bystander, with the exclamation, "All the way from the top, sir!"

*Injuries.—Left hand:* three fingers and the ball of the thumb blistered, not severely; skin slightly torn from the little finger. *Right hand:* flesh abraded from the ball of the thumb one inch in length by half an inch in depth; from outside of little finger three quarters of an inch in length by half an inch in depth; next two fingers excoriated. *Neck:* chafed slightly on the side, over the sterno-cleido-mastoid muscle, two inches by three-quarters of an inch.

When asked of what he thought while falling, he said that he thought he should be killed if he could not catch the rope; that the rope was secure from slipping, because of the hook's being fast (he was, as above stated, attempting to disengage the hook when he fell;) he thought he should be mangled and killed; thought of his home and his parents, and *of a great many other things.* The time occupied in falling could not have been more than one and three quarter seconds.

The common expression with regard to one killed in any similar manner, that such an one "never knew what hurt him," would seem to be disproved by this boy's experience.

3. On November 4, 1854, an engineer on the Providence Railroad fell from the locomotive while it was going at the rate of thirty miles an hour. His shoulder was bruised, but there were no fractures, nor any contusions of consequence besides that upon the shoulder.

Dr. J. B. S. Jackson, referring to the retaining of consciousness at the time of falling from a height, said that he had often been assured by persons thus falling that they had not been sensible of falling.

Dr. C. E. Ware mentioned having once fallen from a height of twenty or thirty feet; he was conscious while falling, and thought of the probable consequences. Dr. W. mentioned another instance: a young man fell forty-five feet from a belfry to the ground, striking upon a wooden pump-handle, and breaking it short off. On recovering from the insensibility resulting from the blow, he was not sensible of remembering anything relative to falling; he recollected only leaving his house just before ascending the belfry.

Dr. Coale thought it certain that *some time* is necessary in order that impressions may be communicated to the brain; if a person be *stunned*, although there may have been an impression of occurrences immediately previous, these will be obliterated by the concussion received. Dr. C., however, related a remarkable case, in which a sailor was conscious, during a fall of about seventy feet from the topsail yard of a frigate. He was aware of striking the foretop in his descent. It is to be remarked, that in cases of falling a short distance only, as from a chair to the floor, if unconsciousness be produced, the obliteration of memory in the person, as to the circumstances, is usually complete.

Dr. PUTNAM knew of a child, five years old, who, while on his way down a flight of stairs in search of a custard which had been promised to him, fell, and sustained fracture of the skull, was unconscious for some days, and lost a piece of bone from the skill; on recovery of consciousness, his first question was in reference to the custard.

Dr. PARKMAN remarked, that where compression of the brain exists, the above is always true of patients; the last idea previous to the accident being the first presenting itself on recovery.

Dr. PUTNAM added, that trephining was contemplated in his patient's case, but was not found necessary.—*American Journal Medical Sciences.*

